# Supplementary material for: Blood glucose fluctuation and in-hospital mortality among patients with acute myocardial infarction: eICU collaborative research database
Source: PLoS One. 2024 Apr 26;19(4):e0300323. doi: 10.1371/journal.pone.0300323 (PMC11051610; doi:10.1371/journal.pone.0300323)
Supplement: S1 Table — (DOCX) [file pone.0300323.s001.docx]

**Supplemental Table 1 Sensitivity analysis of before and after interpolation**

| **Variables** | **Before interpolation (n=2590)** | **After interpolation (n=2590)** | ***P*** |
| --- | --- | --- | --- |
| Height, cm, Mean ± SD | 169.67 ± 10.42 | 169.65 ± 10.42 | 0.950 |
| Weight, kg, Mean ± SD | 83.83 ± 21.45 | 83.88 ± 21.40 | 0.933 |
| Heart rate, beats/minute, Mean ± SD | 88.85 ± 22.54 | 88.69 ± 22.45 | 0.803 |
| Respiratory rate, BPM, M (Q_1_, Q_3_) | 20.00 (16.00, 24.00) | 20.00 (16.00, 24.00) | 0.906 |
| SBP, mmHg, Mean ± SD | 126.39 ± 29.74 | 126.44 ± 29.49 | 0.957 |
| DBP, mmHg, Mean ± SD | 72.29 ± 19.38 | 72.30 ± 19.22 | 0.983 |
| Temperature, ℃, Mean ± SD | 37.00 ± 4.80 | 36.98 ± 4.71 | 0.884 |
| Hemoglobin, g/dL, Mean ± SD | 12.32 ± 2.57 | 12.32 ± 2.57 | 0.961 |
| WBC, K/mcL, M (Q_1_, Q_3_) | 11.80 (8.90, 15.80) | 11.73 (8.90, 15.80) | 0.885 |
| Platelets, K/mcL M (Q_1_, Q_3_) | 217.00 (168.00, 273.00) | 217.00 (168.00, 274.00) | 0.895 |
| RDW, %, Mean ± SD | 14.70 ± 1.98 | 14.70 ± 1.97 | 0.964 |
| Bicarbonate, mmol/L, Mean ± SD | 23.37 ± 4.96 | 23.38 ± 4.93 | 0.911 |
| Sodium, mmol/L, Mean ± SD | 137.43 ± 5.02 | 137.42 ± 5.01 | 0.948 |
| Potassium, mmol/L, Mean ± SD | 4.19 ± 0.78 | 4.19 ± 0.78 | 0.957 |
| Chloride, mmol/L, Mean ± SD | 102.78 ± 6.36 | 102.77 ± 6.34 | 0.977 |
| Creatinine, mg/dL, M (Q_1_, Q_3_) | 1.16 (0.88, 1.71) | 1.16 (0.88, 1.70) | 0.954 |

SBP=systolic blood pressure; DBP=diastolic blood pressure; WBC=white blood cell count; RDW=red blood cell distribution width.
