# Supplementary material for: Blood glucose fluctuation and in-hospital mortality among patients with acute myocardial infarction: eICU collaborative research database
Source: PLoS One. 2024 Apr 26;19(4):e0300323. doi: 10.1371/journal.pone.0300323 (PMC11051610; doi:10.1371/journal.pone.0300323)
Supplement: S2 Table — (DOCX) [file pone.0300323.s002.docx]

**Supplemental Table 2 Screening of covariates**

| **Variables** | **HR (95%CI)** | ***P*** |
| --- | --- | --- |
| Age | 1.02 (1.01-1.03) | <0.001 |
| Gender |  |  |
| Female | Ref |  |
| Male | 0.95 (0.74-1.21) | 0.654 |
| Ethnicity |  |  |
| African American | Ref |  |
| Asian | 1.00 (0.37-2.69) | 0.997 |
| Caucasian | 1.42 (0.89-2.28) | 0.141 |
| Hispanic | 2.05 (1.00-4.23) | 0.051 |
| Other/Unknown | 0.86 (0.43-1.75) | 0.687 |
| ICU type |  |  |
| CCU-CTICU | Ref |  |
| CSICU | 1.42 (0.86-2.37) | 0.174 |
| CTICU | 0.71 (0.35-1.46) | 0.354 |
| CCU | 1.24 (0.82-1.88) | 0.303 |
| MICU | 0.76 (0.38-1.51) | 0.432 |
| MICU-SICU | 0.99 (0.70-1.41) | 0.975 |
| NICU | 1.11 (0.50-2.48) | 0.797 |
| SICU | 0.62 (0.25-1.58) | 0.319 |
| Congestive heart failure |  |  |
| No | Ref |  |
| Yes | 1.27 (0.98-1.65) | 0.075 |
| Atrial fibrillation |  |  |
| No | Ref |  |
| Yes | 1.07 (0.79-1.47) | 0.653 |
| Hypertension |  |  |
| No | Ref |  |
| Yes | 0.67 (0.48-0.95) | 0.025 |
| Diabetes |  |  |
| No | Ref |  |
| Yes | 0.98 (0.73-1.31) | 0.880 |
| Cardiogenic shock |  |  |
| No | Ref |  |
| Yes | 2.42 (1.85-3.17) | <0.001 |
| Sepsis |  |  |
| No | Ref |  |
| Yes | 1.33 (1.02-1.74) | 0.034 |
| BMI | 0.99 (0.98-1.01) | 0.465 |
| Heart rate | 1.01 (1.01-1.01) | 0.042 |
| SBP | 0.99 (0.99-0.99) | 0.003 |
| DBP | 0.99 (0.99-1.00) | 0.122 |
| Respiratory rate | 1.02 (1.01-1.03) | 0.006 |
| Temperature | 1.01 (0.99-1.03) | 0.360 |
| WBC | 1.02 (1.01-1.03) | <0.001 |
| Platelets | 1.00 (1.00-1.00) | 0.305 |
| Hemoglobin | 0.98 (0.93-1.03) | 0.365 |
| RDW | 1.08 (1.03-1.14) | 0.002 |
| Creatinine | 1.08 (1.02-1.15) | 0.006 |
| Bicarbonate | 0.97 (0.95-0.99) | 0.013 |
| Sodium | 1.03 (1.01-1.05) | 0.031 |
| Potassium | 1.18 (1.04-1.35) | 0.014 |
| Chloride | 1.01 (0.99-1.03) | 0.393 |
| Mechanical ventilation |  |  |
| No | Ref |  |
| Yes | 2.48 (1.89-3.24) | <0.001 |
| Vasopressor use |  |  |
| No | Ref |  |
| Yes | 2.48 (1.94-3.18) | <0.001 |
| PCI |  |  |
| No | Ref |  |
| Yes | 1.16 (0.83-1.62) | 0.374 |
| CABG |  |  |
| No | Ref |  |
| Yes | 0.34 (0.18-0.66) | 0.002 |
| Thrombolysis |  |  |
| No | Ref |  |
| Yes | 2.21 (0.98-4.97) | 0.055 |
| Insulin |  |  |
| No | Ref |  |
| Yes | 1.02 (0.78-1.34) | 0.867 |
| ACE inhibitor |  |  |
| No | Ref |  |
| Yes | 0.78 (0.40-1.52) | 0.461 |
| ARB |  |  |
| No | Ref |  |
| Yes | - | 0.967 |
| Anticoagulant administration |  |  |
| No | Ref |  |
| Yes | 0.94 (0.70-1.25) | 0.657 |
| Antiplatelet agent |  |  |
| No | Ref |  |
| Yes | 0.95 (0.73-1.25) | 0.731 |
| Beta blocker |  |  |
| No | Ref |  |
| Yes | 0.71 (0.49-1.04) | 0.079 |
| Calcium channel blocker |  |  |
| No | Ref |  |
| Yes | 0.95 (0.30-2.98) | 0.930 |

ICU=intensive care unit; CCU=coronary heart disease intensive care unit; CTICU= cardiothoracic intensive care unit; CSICU=cardiac surgery intensive care unit; MICU= medical intensive care unit; SICU=surgical intensive care unit; NICU= neurosurgical intensive care unit; BMI=body mass index; SBP=systolic blood pressure; DBP=diastolic blood pressure; WBC=white blood cell count; RDW=red blood cell distribution width; eGFRCKD-EPI =estimated glomerular filtration rate by chronic kidney disease epidemiology collaboration equation; CV=coefficient of variation; PCI=percutaneous coronary intervention; CABG=coronary artery bypass graft; ACE=angiotensin converting enzyme; ARB= angiotensin II receptor blocker.
